# Worldwide burden and epidemiological trends of tracheal, bronchus, and lung cancer: A population-based study

**DOI:** 10.1016/j.ebiom.2022.103951

**Published:** 2022-03-18

**Authors:** Bolun Zhou, Ruochuan Zang, Moyan Zhang, Peng Song, Lei Liu, Fenglong Bie, Yue Peng, Guangyu Bai, Shugeng Gao

**Affiliations:** Thoracic Surgery Department, National Cancer Center/National Clinical Research Center for Cancer/Cancer Hospital, Chinese Academy of Medical Sciences and Peking Union Medical College, Beijing, China

**Keywords:** Epidemiology, Trend analysis, Lung cancer, Global, ASR

## Abstract

**Background:**

We comprehensively analyzed the global burdens and trends in incidence and mortality of tracheal, bronchus, and lung (TBL) cancer among subgroups of distinctive ages and genders.

**Methods:**

We retrieved incidence and mortality rates of lung cancer in 2020 from the GLOBOCAN database among 185 countries. The incidence and mortality age-standardized rates (ASRs) were mostly obtained from Cancer Incidence in Five Continents and World Health Organization mortality database, respectively. The joinpoint regression analysis has been conducted to evaluate the average annual percentage change of incidence and mortality in recent years.

**Findings:**

Trends in the incidence and mortality were decreasing among men in most countries, whereas the trends were increasing among women in some regions. As for mortality, most countries had a decreasing trend in mortality among males, but increasing trends were observed in more than half of countries among females. Furthermore, the majority of countries showed a significant decrease in incidence among males (AAPCs, -0·34 to -6·53), whereas most countries had a significant increase among females (AAPCs, 9·39 to 0·6), especially in European countries. In addition, a more drastic decrease was identified in the trends of the incidence among young people. 33 countries had a drastic decrease among males, especially in countries in Europe (AAPCs, -0·93 to -11·71). And 15 countries showed a significant decrease in incidence among young women (AAPCs, -0·94 to -9·35).

**Interpretation:**

Decreasing incidence and mortality trends were identified in TBL cancer, particularly among all-age men and women younger than 50 years old. But some other groups of individuals showed an opposite trend, such as women in European countries. More preventive interventions are required for the specific populations.

**Funding:**

A full list of funding bodies that contributed to this study can be found in the Acknowledgements section.

**Research in context**

**Evidence before this study**

The tracheal, bronchus, and lung cancer is still one of the most prevalent cancers around the world, and investigating the recent trends in incidence and mortality is crucial for the making of public health policy. We searched PubMed to identify relevant publications up to Sept 12, 2021, and the Institute for Health Metrics and Evaluation's research articles database up to Sept 15, 2021, for the global burden of tracheal, bronchus, and lung cancer using the terms (“lung cancer” OR “bronchus cancer” OR “tracheal cancer” OR “respiratory cancer”) AND (“burden” OR “trend”), with no language restrictions. We have retrieved three major studies related to the global prevalence of TBL cancer. However, these studies were only based on estimated data instead of real-world data from regional cancer registries. Moreover, the precise trends in incidence and mortality for each country and recent trends among people of different age subgroups were still unclear.

**Added value of this study**

In the present study, we have provided updated global and regional burden of lung cancer from GLOBOCAN 2020 database. Furthermore, based on the data from multiple regional registries, we have evaluated ten-year incidence and mortality trends of TBL cancer stratified by age and gender for each country. We have found that recent trends in incidence and mortality of TBL cancer vary from country to country, men to women, and the young to the old. The increasing trends in mortality were observed in most European countries among women, which was totally different from trends among men. And increasing trends in incidence were identified mostly in Asia, Europe and Oceania among women, but the majority of countries had decreasing incidence trends among young women except Asian countries. Finally, we identified the majority of countries with lower HDI had lower incidence and mortality rates than other countries.

**Implications of all the available evidence**

TBL cancer is still regarded as one of the serious public health challenges around the world. Our study has provided comprehensive evidence for health policy makers to guide targeted interventions, aiming to reduce the global burden of TBL cancer. Future studies are supposed to further investigate specific reasons behind these epidemiologic transitions, which might reveal the potential etiology of TBL cancer.

Alt-text: Unlabelled box

## Introduction

Globally, tracheal, bronchus, and lung cancer (TBL) is the second most common cancer and has been ranked as the top cause of cancer-related mortality in 2020, accounting for approximately 2·2 million new cases and almost 1·8 million deaths.^1^ According to the Cancer Tomorrow online tool of the GLOBOCAN, the incident cases are estimated to increase by around 64% by 2040, whereas death cases are estimated to increase by about 67%.^1^ A recent study has revealed that smoking is still the primary risk factor for TBL cancer, and other risk factors include air pollution, exposure to asbestos and high fasting plasma glucose.^2^^,^^3^ The survival rate of TBL cancer is relatively poor and it is still regarded as a substantial public health burden.^4^ And early screening has a great potential for decreasing the mortality of tracheal, bronchus, and lung cancer.^5^^,^^6^ Herein, evaluating the worldwide trends of TBL cancer is helpful to direct future strategies in cancer prevention and treatment.

Due to significant variation of epidemiology among multiple regions or populations, it is critical to investigate the worldwide patterns and temporal trends for the recent period, which could foster health policy formulation and decision making. Previous studies have demonstrated the incidence and mortality rate of TBL in different continents or groups of countries determined by their Socio-demographic Index, suggesting that TBL cancer is more prevalent among countries with higher Socio-demographic Index.^2^ Another study has revealed the differences in incidence and death trends of TBL cancer in various specific regions.^7^ Moreover, Sung et al. have demonstrated that the trends of cancer in younger people were quite different from the elder population in the USA.^8^ And investigating the trends of TBL cancer in the population of different age subgroups is quite crucial, which could accurately assign allocation of resources for people of different ages. However, all the previous studies were based on the Global Burden of Disease (GBD) Study 2019, which examined the incidence and mortality trends in TBL cancer according to estimation and modeling instead of data from regional registries.^9^ Furthermore, the exact incidence and mortality trends at a country level and recent trends in populations of different age and gender subgroups remained largely unknown.

In this study, we used the real-world data of 185 cancer registries to evaluate the most recent epidemiologic trends in the incidence and mortality of TBL cancer. We also analyzed temporal trends among subgroups of different ages and sexes, which could contribute to the making of specific health policies and reduce the cancer global burden in the future.

## Methods

### Data retrieval

The methodological approach used in this study was similar to the recent study investigating the mortality and incidence of pancreatic cancer.^10^ Our study focused on analyzing the worldwide burden of TBL cancer (ICD-10 C33-34) in 2020 and evaluating the incidence and mortality trends over ten years. We obtained the incidence and mortality estimates of lung cancer for 185 countries in 2020 via GLOBOCAN database.^1^ The worldwide population data were extracted from the 2019 Revision of World Population Prospects from the United Nations (UN) population estimates.^11^ The human development index (HDI) data were retrieved from the UN database, which contained the gross national income, expected and mean years of schooling and life expectancy.^12^ According to the quartiles of the distributions, we divided countries into different HDI subgroups (Low: < 0·550; Medium: 0·550 to 0·699; High: 0·700 to 0·799; Very High: > 0·799).^13^

To acquire the incidence data of tracheal, bronchus, and lung cancer for trends analysis, we used the Cancer in Five Continents plus (CI5plus) database, which contained annual incidence data of different countries.^14^ In addition, the incidence data of countries of North Europe were obtained from the Nordic Cancer Registries (NORDCAN)^15^ and data of the United States were obtained from the Surveillance, Epidemiology, and End Results (SEER) Program.^16^ We have obtained the most recent publicly available data and the range of years may vary from country to country. We investigated the incidence data of 46 countries in this study, of which 26 countries had the national incidence data. And for the rest 20 countries without national incidence data, the data from regional registries were used in the study. As for the mortality estimates, we got access to the data via the WHO mortality database.^17^ The mortality data with quality at a medium level or above were included in the WHO mortality database. Also, mortality data of countries of northern Europe and the United States were retrieved from the NORDCAN and SEER database, respectively (Table S1). Because the databases used in the study are available for the public, the ethical approval has been already finished by the committee of each database andadditional ethical approval was not required. No human participant was involved and the informed consent was waived.

### The outcomes and statistical analysis

According to the Segi-Doll world standard population, the incidence and mortality data were adjusted by age and analyzed as the age-standardized rates (ASRs).^18^ Then the joinpoint regression analysis was applied to evaluate the recent ten-year incidence and mortality trends, respectively.^19^ We have excluded “missing” or “zero” value in the analysis because the joinpoint regression could not analyze it. A logarithmic transformation of the incidence and mortality ASRs was conducted and the binomial approximation was used to calculate standard errors. Based on a previous study, we set three as the maximum number of joinpoints in the analysis.^20^ We used the geometric weighting in populations of different countries, sex and age group to calculate the average annual percentage change (AAPC) and the corresponding 95% confidence interval (CI), reflecting the ten-year incidence and mortality trends. We assigned weights equal to the length of each segment to the specified time interval. And the detailed formula of AAPC was: AAPC = $\left\{\exp(\frac{\sum wibi}{\sum wi})-1\right\}$ x 100. The w_i_s represents the length of each segment in the range of years, and the b_i_s denotes the slope coefficients for each segment in the desired range of years. To generate the statistical significance, the magnitude of AAPC was compared with zero and AAPC was significantly different from zero at the alpha = 0.05 level. We repeatedly applied the permutation test for testing between two different joinpoint models. *P* < 0·05 was considered statistically significant and insignificance was considered as a stable trend.

### Role of funding source

The funders played no role in study design, in the collection, analysis and interpretation of data, in the writing of the manuscript and in the decision to submit the paper for publication.

## Results

### Incidence and mortality patterns of Lung cancer in 2020

**Figure 1:**
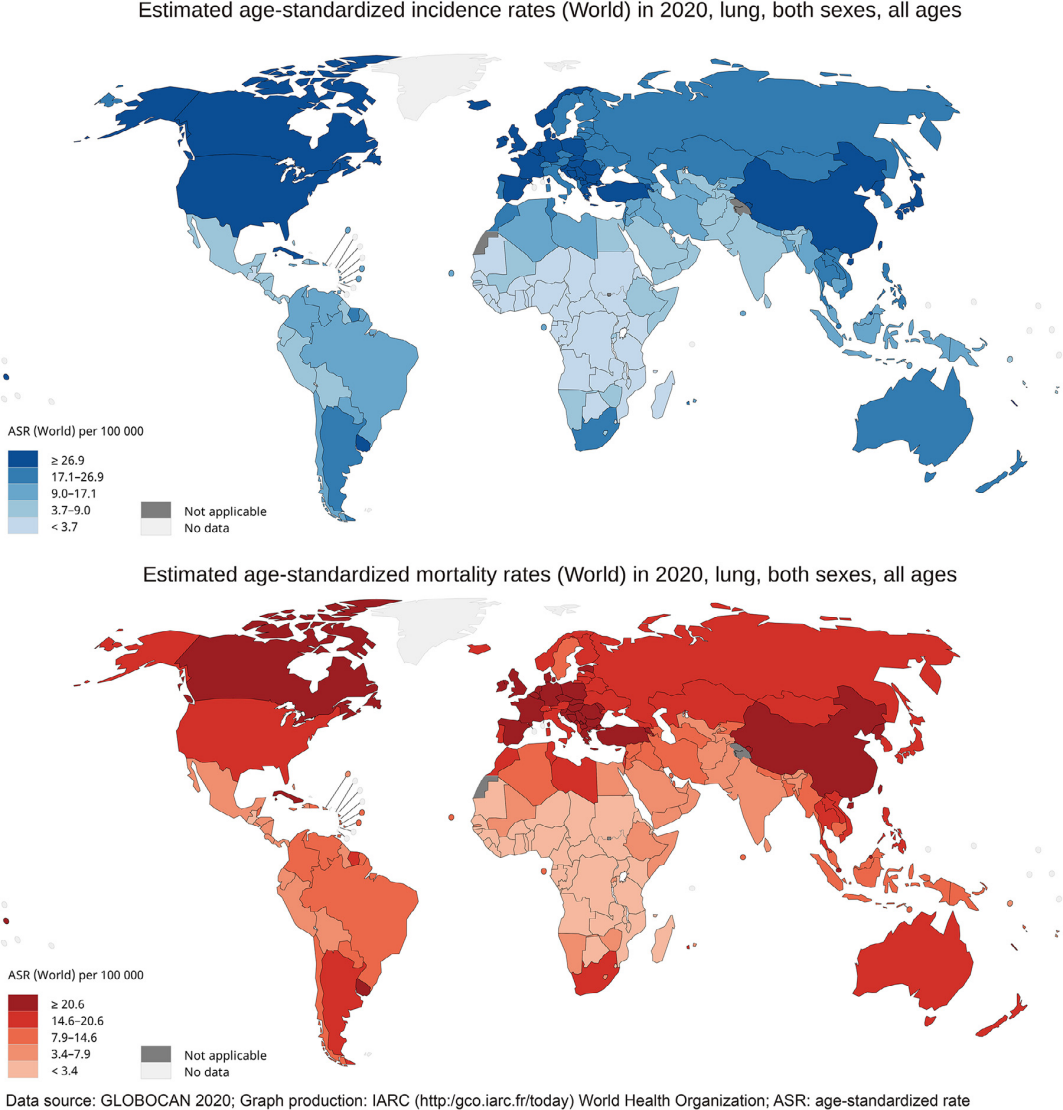
The global estimated incidence and mortality of lung cancer in 2020, both sexes, all ages. The map was produced by the International Agency for Research on Cancer (IARC) and World Health Organization (WHO). The materials and names provided in this study do not represent any opinion of WHO or IARC regarding the legal status of any country, territory, city or region or its authorities or the delimitation of its borders. Dashed lines were used to characterize approximate borderlines that may not yet be full agreement.

In 2020, there were a total 2206771 new cases of lung cancer all over the world. The average incidence ASR was 22·4 per 100000 globally, ranging from 50·1 (Hungary) to 0·88 (Niger). As for the continental level, Eastern Asia (ASR, 34·4), Western Europe (ASR, 32·7) and Northern America (ASR, 32·6) had the top three highest incidence rates. The highest incidence rates were observed in countries with very high HDI (ASR, 29·9), whereas countries with low HDI had the lowest incidence rates (ASR, 3·5). The incidence rate of men (ASR, 31·5) was more than twice as much as the incidence rate of women (ASR, 14.6) globally. As for the mortality rates, the average mortality ASR was 18·0 per 100000 globally, ranging from 42·4 (Hungary) to 0·86 (Niger). And 1796144 people died due to lung cancer around the world in 2020. The highest mortality rates were in Eastern Asia (ASR, 28·1), Western Europe (ASR, 23·8), and Central and Eastern Europe (ASR, 22·7). Mortality rates of countries with very high HDI (ASR, 22·8) were seven times greater than the mortality rate of countries with a low HDI (ASR, 3·2). The detailed data were presented in Figure 1 and Table 1.

**Table 1:** The incidence and mortality of lung cancer in different regions.

	Incidence						Mortality					
	Both sexes		Male		Female		Both sexes		Male		Female	
Regions	Cases	ASR per 100000	Cases	ASR per 100000	Cases	ASR per 100000	Cases	ASR per 100000	Cases	ASR per 100000	Cases	ASR per 100000
World	2206771	22.4	1435943	31.5	770828	14.6	1796144	18	1188679	25.9	607465	11.2
Africa												
Eastern Africa	7419	3.5	3970	4.2	3449	3	6758	3.2	3611	3.9	3147	2.7
Middle Africa	2037	2.5	1270	3.4	767	1.8	1897	2.4	1184	3.2	713	1.7
Northern Africa	23179	11.1	19310	19.5	3869	3.5	20728	9.9	17295	17.5	3433	3.1
Southern Africa	9178	16.9	6283	27.5	2895	9.3	7939	14.6	5408	23.6	2531	8.1
Western Africa	4175	2.2	2449	2.8	1726	1.8	3849	2.1	2262	2.6	1587	1.6
Americas and Caribbean												
Caribbean	11058	17.6	6670	23	4388	13	10079	15.8	6230	21.3	3849	11.1
Central America	9934	5.2	5798	6.7	4136	4	9236	4.8	5499	6.4	3737	3.6
Northern America	253537	32.6	129086	35.7	124451	30.1	159641	19.3	83945	22.2	75696	16.9
South America	76609	13.6	44878	17.8	31731	10.3	67312	11.8	39106	15.4	28206	9.1
Asia												
Eastern Asia	1012021	34.4	670827	48.1	341194	22.1	841174	28.1	558235	39.7	282939	17.8
South-Central Asia	121369	6.6	88130	9.7	33239	3.5	109356	5.9	79 920	8.8	29436	3.1
South-Eastern Asia	123309	17.2	85795	26.4	37514	9.6	109520	15.3	76521	23.7	32999	8.4
Western Asia	58437	24.2	47146	41.7	11291	8.7	52467	21.9	42542	38.3	9925	7.6
Europe												
Northern Europe	75051	29.7	39413	33.3	35638	26.8	54421	20.1	29072	23.2	25349	17.5
Southern Europe	104391	28.7	74009	43.1	30382	16.4	85635	21.9	61692	33.8	23943	11.8
Western Europe	146460	32.7	89646	41.7	56814	25	113524	23.8	72486	32.1	41038	16.7
Central and Eastern Europe	151632	26.9	111986	49	39646	11.6	130596	22.7	96769	42	33827	9.5
Oceania												
Australia and New Zealand	15587	25.2	8372	28.1	7215	22.7	10791	16.2	6104	19.1	4687	13.7
Melanesia	918	13.1	588	17.4	330	9.2	798	11.5	515	15.4	283	8
Micronesia	202	36.4	129	51.3	73	24.6	194	34.9	126	50.1	68	22.7
Polynesia	268	37.3	188	53	80	21.7	229	31.8	157	44.3	72	19.5
HDI												
High HDI	1047707	26.2	697411	37.1	350296	16.5	918661	22.8	610626	32.5	308035	14.3
Very high HDI	975665	29.9	611867	41.1	363798	20.6	711630	20.6	462513	30	249117	12.9
Medium HDI	165943	8	116316	11.5	49627	4.6	149887	7.2	106011	10.6	43876	4.1
Low HDI	16418	3.5	9713	4.5	6705	2.6	15108	3.2	8987	4.2	6121	2.4

### Trends in mortality

**Figure 2:**
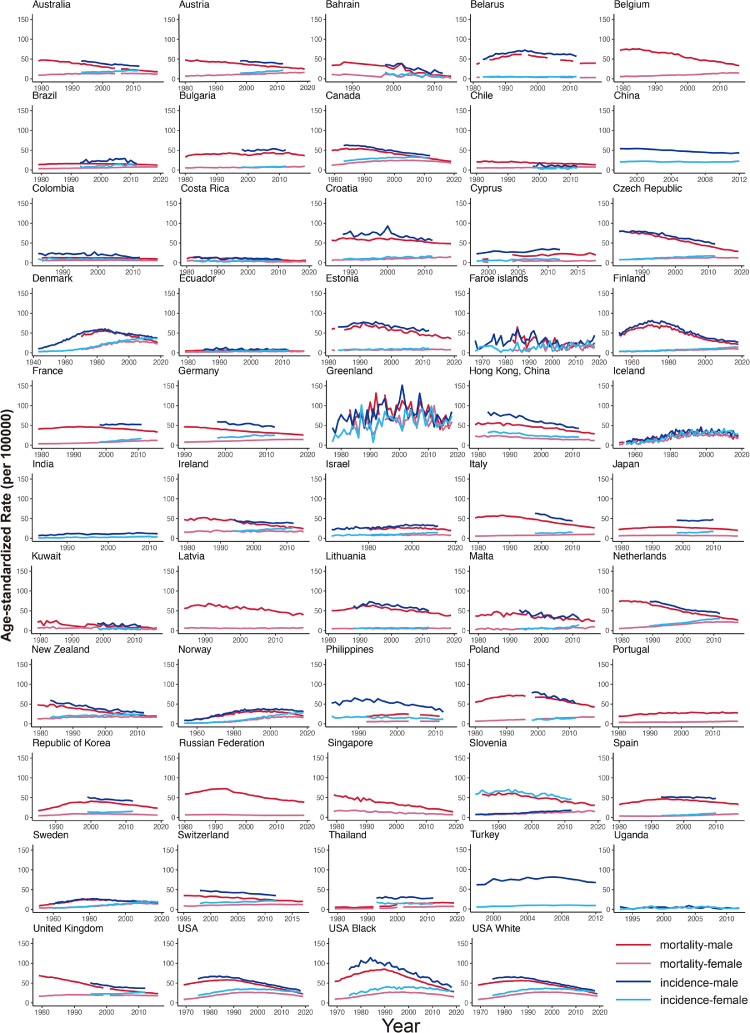
Temporal trends in incidence and mortality of tracheal, bronchus, and lung cancer by country or population in males and females. The dark red lines denote the mortality for males, the light red lines denote the mortality for females. The dark blue lines denote the incidence for males, the light blue lines denote the incidence for females.

**Figure 3:**
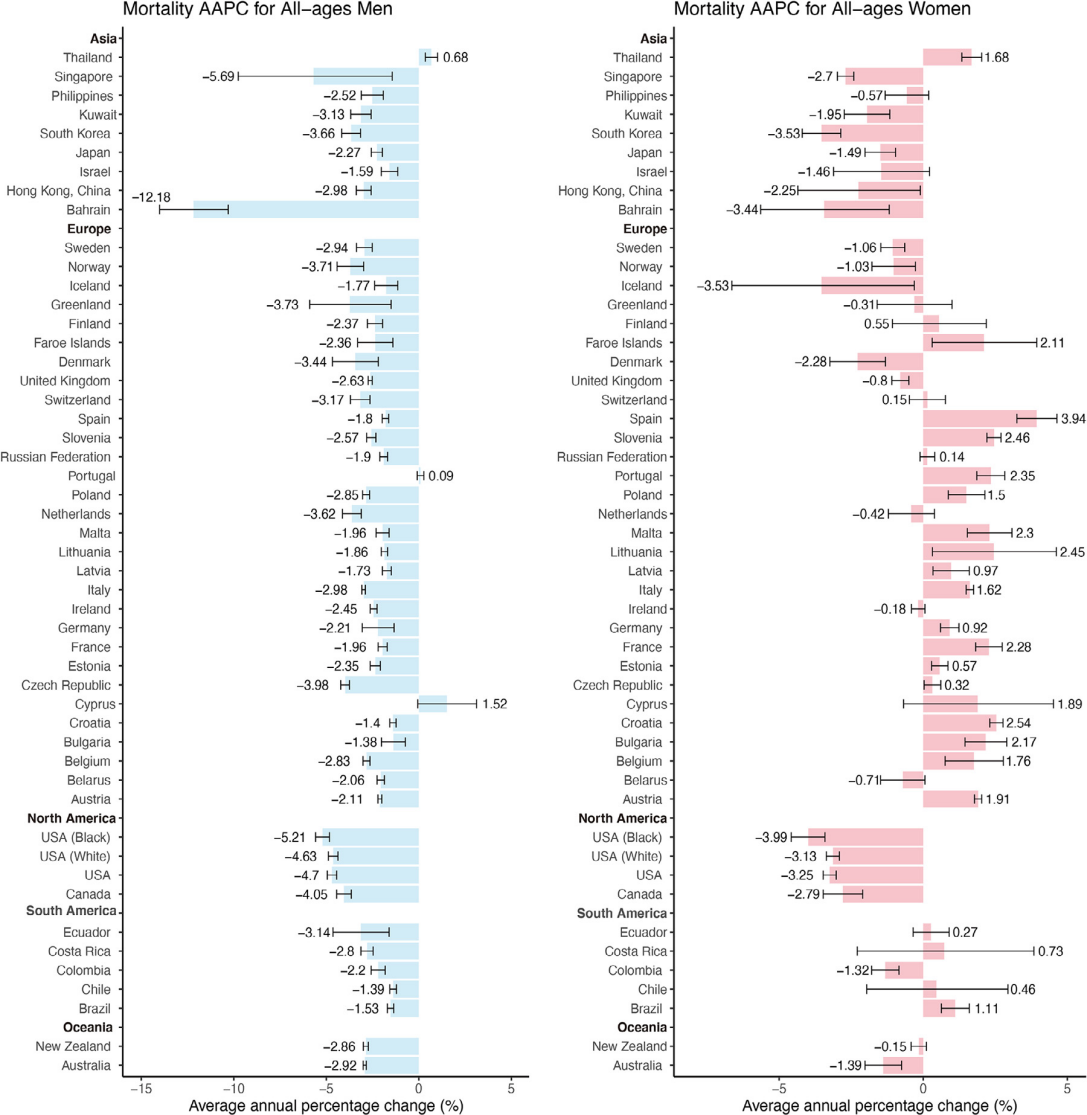
The average annual percentage change (AAPC) of the mortality of tracheal, bronchus, and lung cancer in all-age men and women. The AAPC is denoted by the coloured bars and 95% confidence intervals (CIs) are represented by error bars. The permutation test is applied for testing between two different joinpoint models. *Subnational data.

Nearly all of the countries had a decrease in mortality among males in the past ten years, including all countries in North America and Oceania (AAPCs, -1·38 to -12·18, Figure 3, Table S2). The top three countries or populations with the lowest AAPC were Bahrain (AAPC, -12·18; 95%CI [-14·01, -10·30]), Singapore (AAPC, -5·69; 95%CI [-9·76, -1·44]) and black people in the USA (AAPC, -5·21; 95%CI [-5·58, -4·83]). Only Thailand had a significant increase in mortality among males (AAPC, 0·68; 95%CI [0·35, 1·01]). Among females, 15 countries showed a decreasing trend in mortality (AAPCs, -0·8 to -3·53, Figure 3, Table S2). Countries or populations with the most significant decrease were black people in the USA (AAPC, -3·99; 95%CI [4·58, 3·41]), Iceland (AAPC, -3·53; 95%CI [6·64, 3·31]) and South Korea (AAPC, -3·53; 95%CI [-4·20, -2·86]). And 18 countries showed an increasing trend in mortality, which was different from the trends among males. Compared with other continents, the increase occurred in most of the countries in Europe, such as Spain (AAPC, 3·94; 95%CI [3·25, 4·64]), Slovenia (AAPC, 2·46; 95%CI [2·21, 2·70]) and Lithuania (AAPC, 2·45; 95%CI [0·32, 4·62]). The trends of the mortality ASRs also indicated the same results (Figure 2).

### Trends in incidence

**Figure 4:**
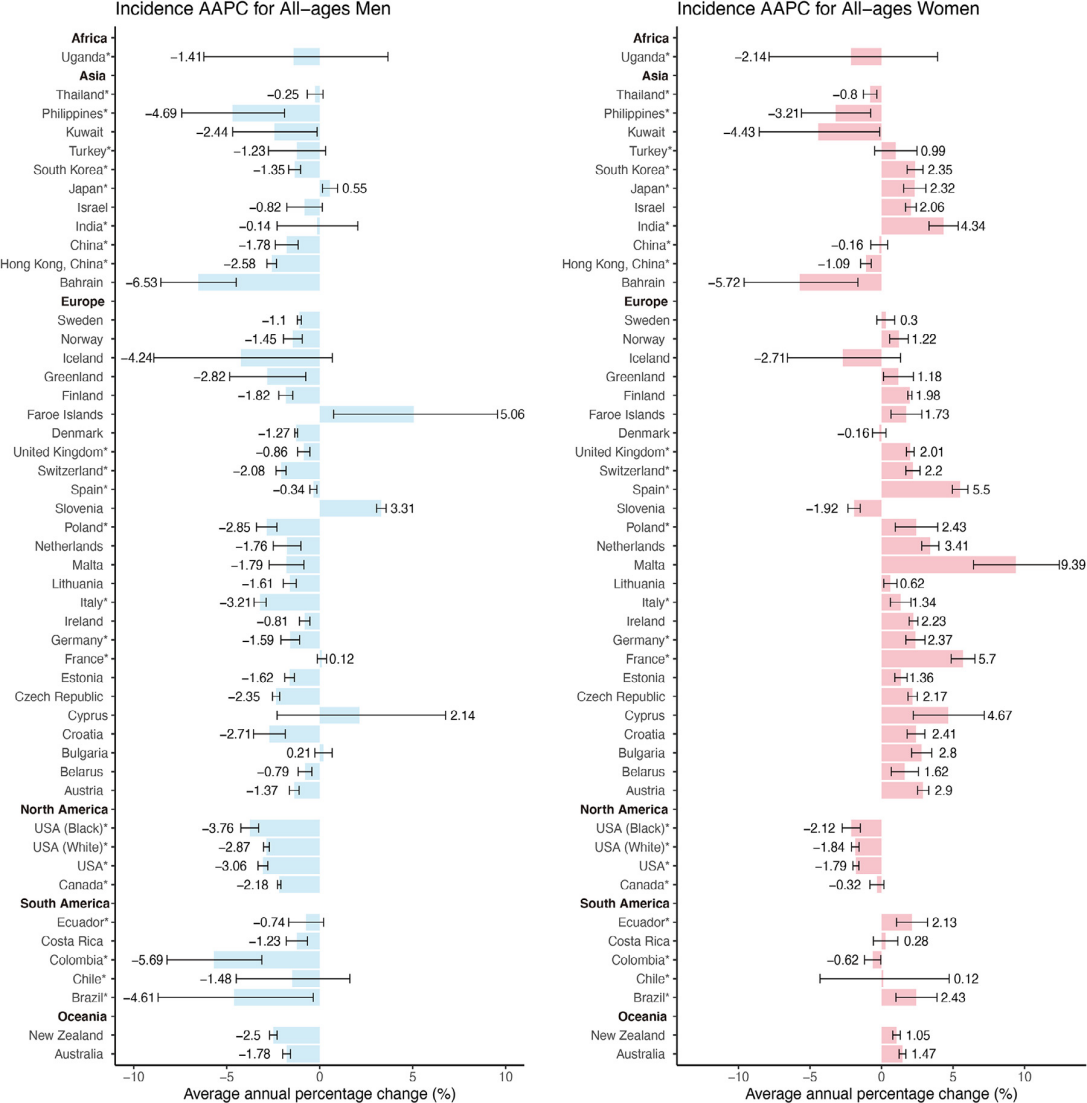
The average annual percentage change (AAPC) of the incidence of tracheal, bronchus, and lung cancer in all-age men and women. The AAPC is denoted by the coloured bars and 95% confidence intervals (CIs) are represented by error bars. The permutation test is applied for testing between two different joinpoint models. *Subnational data.

Among males, the majority of countries (32 of 46) showed a significant decrease in incidence (AAPCs, -0·34 to -6·53, Figure 4, Table S3). Countries with the most significant decrease were Bahrain (AAPC, -6·53; 95%CI [-8·54, -4·49]), Colombia (AAPC, -5·69; 95%CI [-8·21, -3·11]) and Brazil (AAPC, -4·61; 95%CI [-8·69, -0·35]), which were countries in Asia and South America. Only three countries showed a significant increase in incidence among males, including Faroe Islands (AAPC, 5·06; 95%CI [0·75, 9·56]), Slovenia (AAPC, 3·31; 95%CI [3·06, 3·57]) and Japan (AAPC, 0·55; 95%CI [0·15, 0·96]). We also evaluated the incidence trends among males older than 50 years old or younger than 50 years old, which the results showed that similar patterns were more significant among younger males due to the higher AAPCs. Among the younger population, 33 countries had a drastic decrease in males, especially in countries in Europe (AAPCs, -0·93 to -11·71, Fig. S2, Table S5). The top three countries or populations with the lowest AAPC were Colombia (AAPC, -11·71; 95%CI [-20·34, -2·14]), Iceland (AAPC, -10·76; 95%CI [-19·12, -1·54]) and Czech Republic (AAPC, -8·22; 95%CI [-9·01, -7·43]). Seven countries were considered as stable trends and no country had an increase in mortality. A similar trend was observed in the elder population, 35 countries had a significant decrease in incidence, with Bahrain (AAPC, -6·54; 95%CI [-8·58, -4·45]) having the highest decrease, followed by Colombia (AAPC, -5·46; 95%CI [-8·21, -2·62], Fig. S1, Table S4). Only two countries had a significant increase in the elder subgroup, including Japan (AAPC, 0·7; 95%CI [0·24, 1·17]) and Bulgaria (AAPC, 0·62; 95%CI [0·10, 1·15]).

Compared with males, the trends of incidence among females were different. Most countries (30 of 46) had a significant increase in incidence among females (AAPCs, 9·39 to 0·62, Figure 4, Table S3). The increase was found in 21 European countries, which Malta (AAPC, 9·39; 95%CI [6·43, 12·43]), France (AAPC, 5·7; 95%CI [4·87, 6·53]) and Spain (AAPC, 5·5; 95%CI [4·95, 6·04]) were found to show the most significant increasing trends. Countries with the most drastic increase in other continents included India (AAPC, 4·34; 95%CI [3·32, 5·36]), Brazil (AAPC, 2·43; 95%CI [1·01, 3·88]), and South Korea (AAPC, 2·35; 95%CI [1·79, 2·90]). Eight regions showed a decrease in incidence and most were in Asia (5 of 8), including Bahrain (AAPC, -5·72; 95%CI [-9·61,-1·65]), Kuwait (AAPC, -4·43; 95%CI [-8·55, -0·13]) and Philippines (AAPC, -3·21; 95%CI [-5·60, -0·76]). We can draw the same conclusion based on the trends of the incidence ASRs (Figure 2). As for the analysis in the younger and elder subgroups, the trends were different. 15 countries showed a drastic decrease in incidence among younger women, with Poland (AAPC, -9·35; 95%CI [-15·08, -3·23]) having the highest decrease (AAPCs, -0·94 to -9·35, Fig. S2, Table S5). Only 8 countries had an increasing trend, such as Malta (AAPC, 5·79; 95%CI [2·00, 9·73]) and Brazil (AAPC, 4·42; 95%CI [1·16, 7·79]). Compared with the all-age groups, the trend was dramatically opposite, especially among countries in Europe. On the contrary, the majority of countries (28 of 45) had a significant increase in incidence among elder females, which was similar to the pattern of the all-age group (AAPCs, 10·09 to 0·95, Fig. S1, Table S4). Only 5 countries showed a decrease in incidence, with Bahrain having the highest decrease (AAPC, -5·65; 95%CI [-9·37, -1·78]).

### Relationship between ASR and AAPC among countries of different HDI

**Figure 5:**
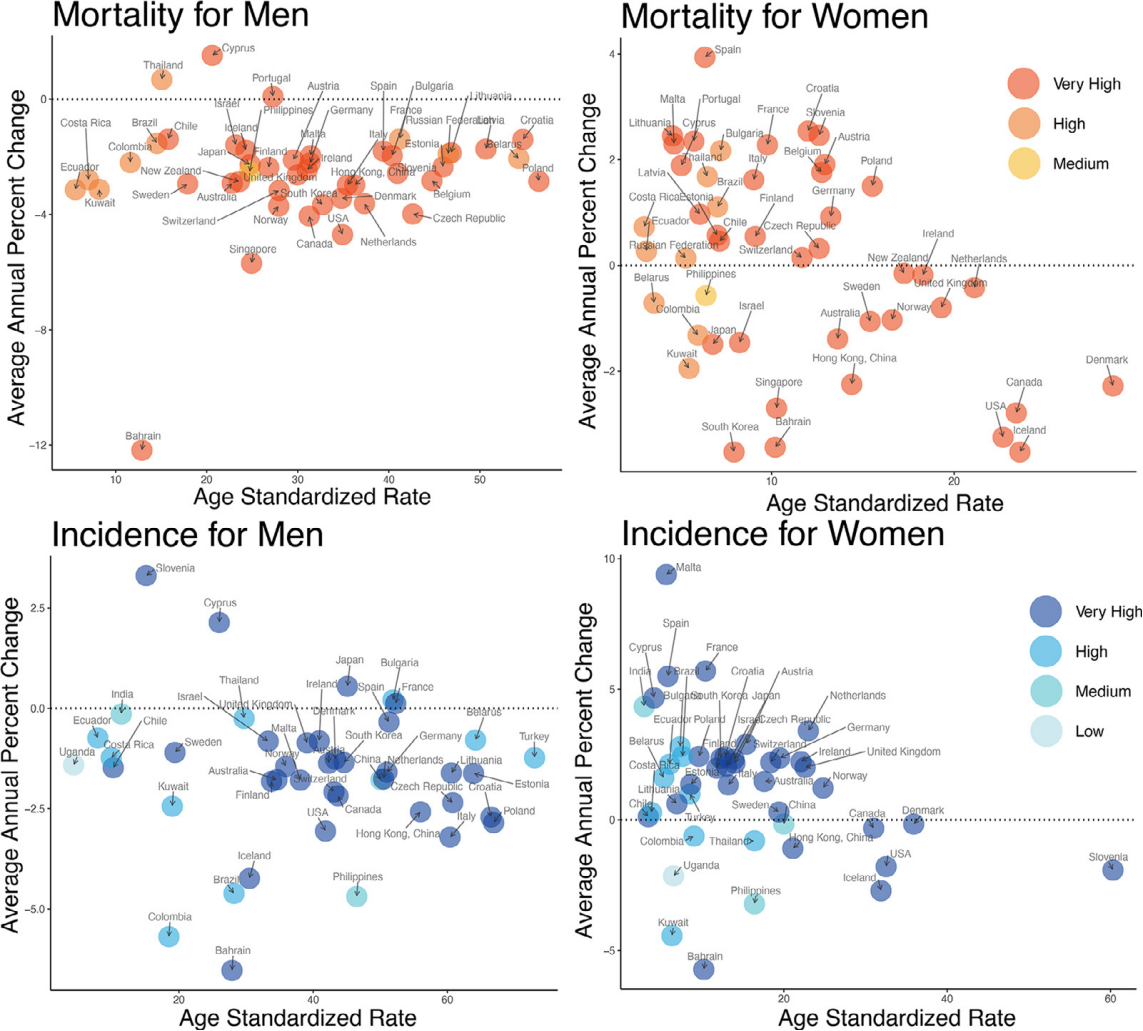
The age standardized rate at the start of each study period versus average annual percentage change during the recent 10 years for tracheal, bronchus, and lung cancer. The red bubbles denote the mortality and the blue bubbles denote the incidence. The red and blue deepen as the human development index (HDI) increases.

We also compared the ASR at the beginning of each study period with the AAPC, in which the results varied from males to females (Figure 5). As for mortality, the majority of countries with lower HDI had lower ASRs among males and females. The AAPCs of most countries did not correlate with the ASRs and the greatest AAPCs were identified for Cyprus and Thailand among males. In contrast, the greatest and the lowest AAPCs were identified for Spain and South Korea (two countries with very high HDI) among females, respectively. As for the incidence, Bahrain with a very high HDI had the lowest AAPC in both males and females. Slovenia had the greatest AAPC among males and the AAPC of Malta was the highest among females, which were both belonged to the countries with very high HDI. And most countries with a lower HDI had a lower ASR, which was especially significant in females. Furthermore, countries with the greatest ASR were presented a declining AAPC during the ten-year period, which was similar to the trends of mortality.

## Discussion

Previous studies have focused on the incidence or mortality in some countries, such as England,^21^ Belgium,^22^ Brazil,^23^ China and USA.^24^ However, a comprehensive evaluation of the most updated incidence and mortality trends according to gender and age is lacking. In the present study, we evaluated the real-world population data of regional registries on a global scale, revealing the global patterns and trends in incidence and mortality of TBL cancer. We found that^1^ a substantial number of countries with very high HDI had higher incidence and mortality ASRs in both men and women than other countries^2^; decreasing trends in mortality were found in the males of almost all countries, whereas females of nearly half of the countries had an increasing trend in mortality^3^; decreasing trends in incidence were found among males of the majority of countries, especially for men younger than 50 years old^4^; females of most countries were identified to have an increasing trend in incidence, while females younger than 50 years exhibited a quite opposite trend.

Significant disparities were identified in the burden of TBL cancer among different countries. Our results revealed that most countries with very high HDI had relatively higher incidence and mortality ASRs, while other countries had lower ASRs, which was similar to the results of previous publications. Countries with very high HDI had a higher proportion of elders and a more severe air population than other countries, which could contribute to a higher prevalence of TBL cancer.^25^^,^^26^ Furthermore, people in countries with very high HDI have higher health awareness and could get access to advanced screening methods more easily. Herein, in countries with very high HDI, more cases could be identified even before symptoms onset due to the improved methods to diagnose. For example, after the implementation of LDCT screening in Shanghai, one of the most developed cities in China, the incidence of females with lung cancer rose significantly.^27^ In addition, the mortality and incidence rates of countries with lower HDI may also be underestimated because of the non-functionality of the public health system.

Males in nearly all countries have had decreasing incidence and mortality trends in recent years, whereas the trends varied in females among different countries. The significant decrease in the incidence and mortality of TBL cancer may reveal a declining prevalence of risk factors. The widespread application of LDCT screening could result in an increased incidence of early TBL cancer diagnosis and a corresponding decrease in TBL cancer mortality. For example, the LDCT-based screening program has been introduced in the United States for the past decades, and we observed a significant decrease in the mortality trends among men and women.^5^^,^^28^ However, Gao et al. have proposed that the LDCT-based screening program of most Asian women was associated with considerable lung cancer overdiagnosis.^29^ According to our results, the increasing incidence of TBL cancer among women was not entirely driven by an increase in TBL cancer diagnoses, and the LDCT-based screening program may affect the trends. Compared with the study of Gao et al., far more people and regions were involved in our study and we believed that our results may be more applicable globally. The use of targeted therapies may also result in a sharp fall in mortality in some countries, such as the United States.^30^ An evident increase in incidence was found among females of almost all countries, which may be attributed to levels of hormones. Previous studies have demonstrated that the use of the combined hormone replacement therapy (estrogen plus progestin) could lead to a fifty percent higher risk for women to suffer from lung cancer, indicating the potential role of hormones in carcinogenesis.^31^^,^^32^ Thus, the change of hormones in the past few years may lead to an increasing incidence rate among women. Moreover, increasing trends in mortality were identified in females, especially females from Europe. Smoking is an indisputable risk factor for lung cancer and some research has found that women who smoke could be much more sustainable to lung cancer than men.^33^^,^^34^ According to data from WHO, the smoking rate of women has been increased in Europe and is much higher than the global average rate, which may lead to a substantial increase in mortality rate.^35^

Compared with all age populations and the old, young people have a substantially different trend in incidence, especially among women. However, the exact reasons for the different incidence trends of early-onset lung cancer remain largely uncertain. Previous studies have investigated the potential genetic profiles of early-onset lung cancer, which also revealed that adenocarcinoma was the most prevalent subtype of early-onset lung cancer among young women.^36^ As for males, our results indicated that the decreasing trend in incidence was much more significant among the young than the old. And one recent study has shown that the decreasing trend was appreciably larger in the young men than the old men in Europe.^37^ Furthermore, we found that decreasing trend in incidence was more drastic among young men than young women, which was consistent with a recent publication revealing lung cancer incidence was higher in young women in the United States.^38^ According to data from WHO, smoking prevalence was declining among the young, which was consistent with the decreasing trends in incidence among young people.^39^ In addition, the improvement of health awareness and application of non-smoking norms may also reduce exposure to second-hand smoke. Moreover, the significant decrease in incidence among young people was mostly observed in countries with high income, such as countries in Europe and North America. In most Asian countries, the difference in incidence was not obvious between the young and the old. Due to the close relationship between air pollution and lung cancer incidence, we hypothesized that high air quality in the past few decades may result in a significant decrease in lung cancer incidence among the young from high-income countries.^40^ In contrast, some medium- and low-income countries have developed the economy at the expense of the environment over the past few years, leading to an opposite pattern in incidence that is opposite to those of developed countries.

This study has comprehensively analyzed the geographical variations of incidence and mortality of TBL cancer in 2020 by age and gender, and recent trends in a substantial number of countries. Nevertheless, some limitations still existed. First, the incidence and mortality data may be underestimated in low-income countries due to the limited resources and the suboptimal reporting mechanism. In contrast, data from only one cancer registry in some developed countries may be overestimated because underdeveloped regions of the same country have not been considered. Second, it may be difficult to directly compare the incidence and mortality rate among countries because cancer registries might be different by multiple countries and over time. But the comparison between age subgroups and sex subgroups in the same country may not be influenced. Moreover, due to the inherent limitation of the databases, the periods obtained vary from country to country. Although the most recent publicly available data have been used for the analysis, it still might cause the bias for the comparison among countries. We have used the same strategy of previous publications and varied periods may have very little effect on the results. Third, we have excluded the “missing” or “zero” data in the analysis due to the requirements of the joinpoint regression analysis. The missing data might have a very limited impact on the inferences of this study because only a small fraction of values are missing and individual missing data could hardly affect the trends. Fourth, the recorded incidence and mortality data were still lacking in many countries, especially in undeveloped countries.

We have observed the global trends in incidence and mortality of TBL cancer in the past few years. Although trends in incidence and mortality were decreasing in multiple countries, the total number of people with TBL cancer was still high. More medical resources are needed to cure patients or prolong patients’ survival as much as possible, which may largely affect the socioeconomic situation due to the huge burden of disease. Moreover, health policymakers are supposed to pay more attention to women with this disease, which women have an increasing trend in incidence and mortality, especially in some European countries. In addition, air pollution has been regarded as one of the leading causes of lung cancer, and the balance between environment and economy should be addressed, especially in some developing countries. Future studies are supposed to explore the causes of these epidemiologic transitions, which may reveal the potential etiology of TBL cancer.

## Data sharing statement

Data are available in a public, open-access repository.

## Contributors

BZ and SG designed and supervised the study. BZ, RC, MZ, PS and SG analyzed the data and verified the underlying data. BZ wrote the original draft. LL, FB, YP, GB and SG edited the draft. All authors have read and approved the final manuscript.

## Declaration of interests

All the authors have declared no conflicts of interest and completed the ICMJE forms.

## Appendix Supplementary materials

Image, application 1

Image, application 2
