# Supplementary material for: Worldwide burden and epidemiological trends of tracheal, bronchus, and lung cancer: A population-based study
Source: eBioMedicine. 2022 Mar 18;78:103951. doi: 10.1016/j.ebiom.2022.103951 (PMC8935504; doi:10.1016/j.ebiom.2022.103951)
Supplement: Supplementary file 1 [file mmc1.pdf]

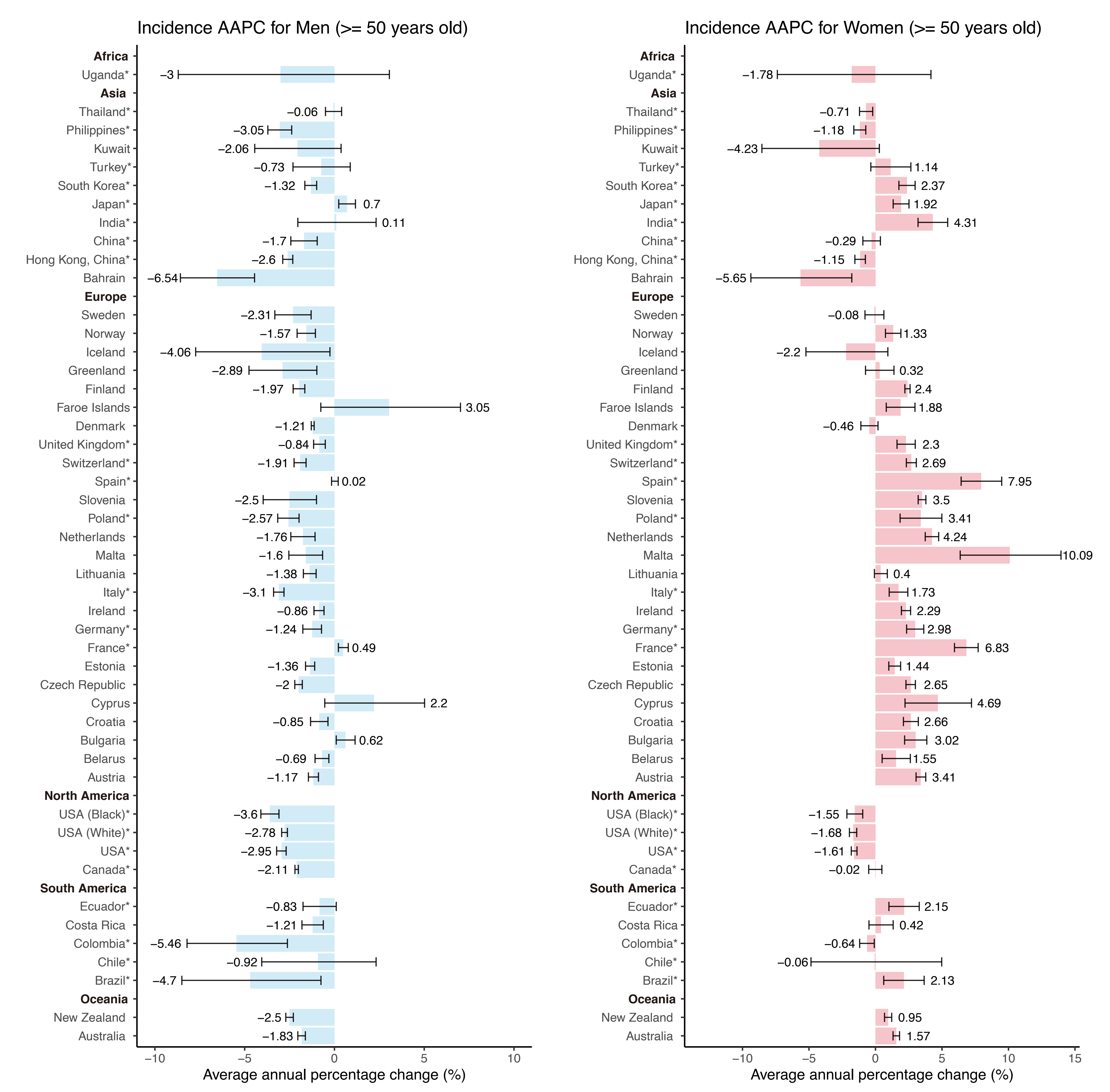
Incidence AAPC for Men (>= 50 years old)
Incidence AAPC for Women (>= 50 years old)
Africa
Uganda*
Asia
Thailand*
Philippines*
Kuwait
Turkey*
South Korea*
Japan*
India*
China*
Hong Kong, China*
Bahrain
Europe
Sweden
Norway
Iceland
Greenland
Finland
Faroe Islands
Denmark
United Kingdom*
Switzerland*
Spain*
Slovenia
Poland*
Netherlands
Malta
Lithuania
Italy*
Ireland
Germany*
France*
Estonia
Czech Republic
Cyprus
Croatia
Bulgaria
Belarus
Austria
North America
USA (Black)*
USA (White)*
USA*
Canada*
South America
Ecuador*
Costa Rica
Colombia*
Chile*
Brazil*
Oceania
New Zealand
Australia
Men: −3, −0.06, −3.05, −2.06, −0.73, −1.32, 0.7, 0.11, −1.7, −2.6, −6.54, −2.31, −1.57, −4.06, −2.89, −1.97, 3.05, −1.21, −0.84, −1.91, 0.02, −2.5, −2.57, −1.76, −1.6, −1.38, −3.1, −0.86, −1.24, 0.49, −1.36, −2, 2.2, −0.85, 0.62, −0.69, −1.17, −3.6, −2.78, −2.95, −2.11, −0.83, −1.21, −5.46, −0.92, −4.7, −2.5, −1.83
Women: −1.78, −0.71, −1.18, −4.23, 1.14, 2.37, 1.92, 4.31, −0.29, −1.15, −5.65, −0.08, 1.33, −2.2, 0.32, 2.4, 1.88, −0.46, 2.3, 2.69, 7.95, 3.5, 3.41, 4.24, 10.09, 0.4, 1.73, 2.29, 2.98, 6.83, 1.44, 2.65, 4.69, 2.66, 3.02, 1.55, 3.41, −1.55, −1.68, −1.61, −0.02, 2.15, 0.42, −0.64, −0.06, 2.13, 0.95, 1.57
−10
−5
0
5
10
15
Average annual percentage change (%)

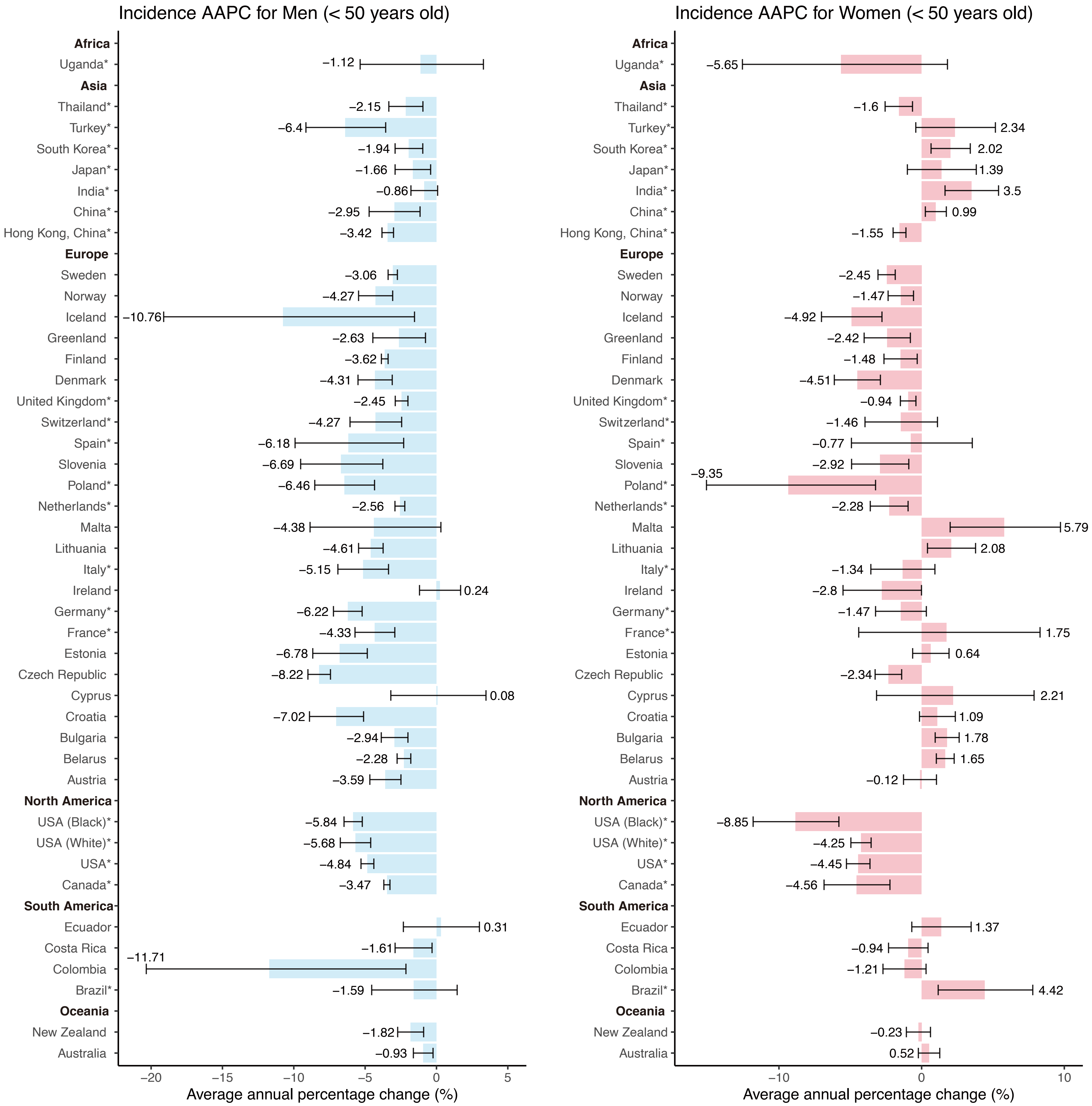
Incidence AAPC for Men (< 50 years old)
Africa
Uganda* −1.12
Asia
Thailand* −2.15
Turkey* −6.4
South Korea* −1.94
Japan* −1.66
India* −0.86
China* −2.95
Hong Kong, China* −3.42
Europe
Sweden −3.06
Norway −4.27
Iceland −10.76
Greenland −2.63
Finland −3.62
Denmark −4.31
United Kingdom* −2.45
Switzerland* −4.27
Spain* −6.18
Slovenia −6.69
Poland* −6.46
Netherlands* −2.56
Malta −4.38
Lithuania −4.61
Italy* −5.15
Ireland 0.24
Germany* −6.22
France* −4.33
Estonia −6.78
Czech Republic −8.22
Cyprus 0.08
Croatia −7.02
Bulgaria −2.94
Belarus −2.28
Austria −3.59
North America
USA (Black)* −5.84
USA (White)* −5.68
USA* −4.84
Canada* −3.47
South America
Ecuador 0.31
Costa Rica −1.61
Colombia −11.71
Brazil* −1.59
Oceania
New Zealand −1.82
Australia −0.93
−20
−15
−10
−5
0
5
Average annual percentage change (%)
Incidence AAPC for Women (< 50 years old)
Africa
Uganda* −5.65
Asia
Thailand* −1.6
Turkey* 2.34
South Korea* 2.02
Japan* 1.39
India* 3.5
China* 0.99
Hong Kong, China* −1.55
Europe
Sweden −2.45
Norway −1.47
Iceland −4.92
Greenland −2.42
Finland −1.48
Denmark −4.51
United Kingdom* −0.94
Switzerland* −1.46
Spain* −0.77
Slovenia −2.92
Poland* −9.35
Netherlands* −2.28
Malta 5.79
Lithuania 2.08
Italy* −1.34
Ireland −2.8
Germany* −1.47
France* 1.75
Estonia 0.64
Czech Republic −2.34
Cyprus 2.21
Croatia 1.09
Bulgaria 1.78
Belarus 1.65
Austria −0.12
North America
USA (Black)* −8.85
USA (White)* −4.25
USA* −4.45
Canada* −4.56
South America
Ecuador 1.37
Costa Rica −0.94
Colombia −1.21
Brazil* 4.42
Oceania
New Zealand −0.23
Australia 0.52
−10
0
10
Average annual percentage change (%)

Table S1. The incidence and mortality data of tracheal, bronchus, and lung cancer

Country	Incidence		Mortality	
Australia	1993-2012	CI5	1979-2018	WHO
Austria	1998-2012	CI5	1980-2019	WHO
Bahrain	1998-2012	CI5	1985-2014	WHO
Belarus	1983-2012	CI5	1981-2018	WHO
Belgium	NA	NA	1979-2016	WHO
Brazil	1993-2012	CI5*	1979-2018	WHO
Bulgaria	1998-2012	CI5	1980-2018	WHO
Canada	1983-2012	CI5*	1979-2019	WHO
Chile	1998-2012	CI5*	1980-2018	WHO
China	1998-2012	CI5*	NA	NA
Colombia	1983-2012	CI5*	1984-2017	WHO
Costa Rica	1982-2011	CI5	1980-2018	WHO
Croatia	1988-2012	CI5	1985-2017	WHO
Cyprus	1998-2012	CI5	1999-2018	WHO
Czech Republic	1983-2012	CI5	1986-2019	WHO
Denmark	1943-2018	Nordcan	1970-2018	Nordcan
Ecuador	1985-2012	CI5*	1979-2017	WHO
Estonia	1983-2012	CI5	1994-2019	WHO
Faroe Islands	1961-2018	Nordcan	1983-2018	Nordcan
Finland	1953-2018	Nordcan	1953-2018	Nordcan
France	1998-2011	CI5*	1979-2016	WHO
Germany	1998-2012	CI5*	1990-2019	WHO
Greenland	1977-2018	Nordcan	1983-2018	Nordcan
China, Hong Kong	1983-2012	CI5*	1979-2017	WHO
Iceland	1955-2018	Nordcan	1951-2018	Nordcan
India	1983-2012	CI5*	NA	NA
Ireland	1994-2012	CI5	1979-2015	WHO
Israel	1963-2012	CI5	1979-2018	WHO
Italy	1998-2010	CI5*	1979-2017	WHO
Japan	1998-2010	CI5*	1979-2017	WHO
South Korea	1999-2012	CI5*	1985-2019	WHO
Kuwait	1998-2012	CI5	1979-2017	WHO
Latvia	NA	NA	1984-2018	WHO
Lithuania	1988-2012	CI5	1993-2019	WHO
Malta	1993-2012	CI5	1979-2017	WHO
Netherlands	1989-2012	CI5	1979-2018	WHO
New Zealand	1983-2012	CI5	1979-2016	WHO
Norway	1953-2018	Nordcan	1961-2018	Nordcan
Philippines	1983-2012	CI5*	1992-2014	WHO
Poland	1998-2012	CI5*	1980-2018	WHO
Portugal	NA	NA	1980-2018	WHO

Russian Federation	NA	NA	1980-2019	WHO
Singapore	NA	NA	1979-2019	WHO
Slovenia	1983-2012	CI5	1985-2019	WHO
Spain	1993-2010	CI5*	1980-2017	WHO
Sweden	1960-2018	Nordcan	1952-2018	Nordcan
Switzerland	1998-2012	CI5*	1995-2017	WHO
Turkey	1998-2012	CI5*	NA	NA
Thailand	1993-2012	CI5*	1979-2019	WHO
Uganda	1993-2012	CI5*	NA	NA
United Kingdom	1995-2012	CI5*	1979-2016	WHO
USA	1975-2018	SEER*	1969-2019	SEER
USA Black	1975-2018	SEER*	1969-2020	SEER
USA White	1975-2018	SEER*	1969-2021	SEER

CI5: Cancer Incidence in Five Continents V; NORDCAN: Nordic Cancer Registries; SEER: Surveillance, Epidemiology, and End Results Program; WHO: World Health Organization; NA: not available.

*Subnational data; Brazil, Goiania; Canada (excl. Nunavut, Quebec and Yukon); Chile, Valdivia; China (5 registries); Colombia, Cali; Ecuador, Quito; France (9 registries); Germany (2 registries); India, Chennai; Italy (8 registries); Japan (4 registries); Korea (5 registries); Republic of Korea; Philippines, Manila; Poland, Kielce; Spain (9 registries); Switzerland (6 registries); Thailand (4 registries); Turkey (2 registries); Uganda, Kampala; UK, England; USA, (9 registries)

Table S2. The AAPC of the mortality of tracheal, bronchus, and lung cancer in individuals of all ages.

Country	Lower endpoint	Upper endpoint	AAPC	95%CI-Low	95%CI-High	P-value	Sex
Asia							
Bahrain	2005	2014	-12.18	-14.01	-10.3	< 0.001	male
Hong Kong, China	2008	2017	-2.98	-3.38	-2.58	< 0.001	male
Israel	2009	2018	-1.59	-2.03	-1.14	< 0.001	male
Japan	2009	2018	-2.27	-2.57	-1.97	< 0.001	male
South Korea	2010	2019	-3.66	-4.17	-3.15	< 0.001	male
Kuwait	2008	2017	-3.13	-3.68	-2.58	< 0.001	male
Philippines	2001	2014	-2.52	-3.11	-1.92	< 0.001	male
Singapore	2009	2019	-5.69	-9.76	-1.44	0.009	male
Thailand	2010	2019	0.68	0.35	1.01	< 0.001	male
Europe							
Austria	2010	2019	-2.11	-2.21	-2	< 0.001	male
Belarus	2002	2018	-2.06	-2.26	-1.86	< 0.001	male
Belgium	2007	2016	-2.83	-3.02	-2.64	< 0.001	male
Bulgaria	2009	2018	-1.38	-2.02	-0.73	< 0.001	male
Croatia	2008	2017	-1.4	-1.57	-1.23	< 0.001	male
Cyprus	2009	2018	1.52	-0.06	3.12	0.057	male
Czech Republic	2010	2019	-3.98	-4.22	-3.75	< 0.001	male
Estonia	2010	2019	-2.35	-2.63	-2.08	< 0.001	male
France	2007	2016	-1.96	-2.2	-1.71	< 0.001	male
Germany	2010	2019	-2.21	-3.06	-1.34	< 0.001	male
Ireland	2006	2015	-2.45	-2.63	-2.26	< 0.001	male
Italy	2008	2017	-2.98	-3.08	-2.89	< 0.001	male
Latvia	2009	2018	-1.73	-1.97	-1.49	< 0.001	male
Lithuania	2010	2019	-1.86	-2.03	-1.69	< 0.001	male
Malta	2008	2017	-1.96	-2.31	-1.61	< 0.001	male
Netherlands	2009	2018	-3.62	-4.13	-3.11	< 0.001	male
Poland	2009	2018	-2.85	-3.04	-2.66	< 0.001	male
Portugal	2009	2018	0.09	-0.09	0.27	0.32	male
Russian Federation	2010	2019	-1.9	-2.11	-1.69	< 0.001	male
Slovenia	2010	2019	-2.57	-2.82	-2.32	< 0.001	male
Spain	2008	2017	-1.8	-1.98	-1.63	< 0.001	male
Switzerland	2008	2017	-3.17	-3.7	-2.64	< 0.001	male
United Kingdom	2007	2016	-2.63	-2.74	-2.52	< 0.001	male
Denmark	2009	2018	-3.44	-4.67	-2.19	< 0.001	male

Faroe Islands	2009	2018	-2.36	-3.31	-1.4	< 0.001	male
Finland	2009	2018	-2.37	-2.78	-1.96	< 0.001	male
Greenland	2009	2018	-3.73	-5.9	-1.5	0.002	male
Iceland	2009	2018	-1.77	-2.39	-1.14	< 0.001	male
Norway	2009	2018	-3.71	-4.42	-2.98	< 0.001	male
Sweden	2009	2018	-2.94	-3.37	-2.52	< 0.001	male
North America							
Canada	2010	2019	-4.05	-4.44	-3.65	< 0.001	male
USA	2010	2019	-4.7	-4.95	-4.44	< 0.001	male
USA (White)	2010	2019	-4.63	-4.89	-4.37	< 0.001	male
USA (Black)	2010	2019	-5.21	-5.58	-4.83	< 0.001	male
South America							
Brazil	2010	2019	-1.53	-1.7	-1.36	< 0.001	male
Chile	2009	2018	-1.39	-1.57	-1.21	< 0.001	male
Colombia	2008	2017	-2.2	-2.58	-1.82	< 0.001	male
Costa Rica	2010	2019	-2.8	-3.13	-2.47	< 0.001	male
Ecuador	2008	2017	-3.14	-4.64	-1.61	< 0.001	male
Oceania							
Australia	2009	2018	-2.92	-3.01	-2.84	< 0.001	male
New Zealand	2007	2016	-2.86	-3	-2.73	< 0.001	male
Asia							
Bahrain	2005	2014	-3.44	-5.64	-1.18	0.005	female
Hong Kong, China	2008	2017	-2.25	-4.35	-0.1	0.04	female
Israel	2009	2018	-1.46	-3.11	0.22	0.088	female
Japan	2009	2018	-1.49	-2.02	-0.96	< 0.001	female
South Korea	2010	2019	-3.53	-4.2	-2.86	< 0.001	female
Kuwait	2008	2017	-1.95	-2.74	-1.16	< 0.001	female
Philippines	2001	2014	-0.57	-1.32	0.19	0.139	female
Singapore	2009	2019	-2.7	-2.98	-2.41	< 0.001	female
Thailand	2010	2019	1.68	1.34	2.03	< 0.001	female
Europe							
Austria	2010	2019	1.91	1.78	2.03	< 0.001	female
Belarus	2002	2018	-0.71	-1.48	0.06	0.07	female
Belgium	2007	2016	1.76	0.76	2.78	0.001	female
Bulgaria	2009	2018	2.17	1.45	2.9	< 0.001	female
Croatia	2008	2017	2.54	2.31	2.77	< 0.001	female
Cyprus	2009	2018	1.89	-0.68	4.52	0.139	female
Czech Republic	2010	2019	0.32	0.03	0.61	0.033	female
Estonia	2010	2019	0.57	0.29	0.86	< 0.001	female

France	2007	2016	2.28	1.82	2.74	< 0.001	female
Germany	2010	2019	0.92	0.6	1.24	< 0.001	female
Ireland	2006	2015	-0.18	-0.41	0.06	0.133	female
Italy	2008	2017	1.62	1.49	1.75	< 0.001	female
Latvia	2009	2018	0.97	0.34	1.6	0.004	female
Lithuania	2010	2019	2.45	0.32	4.62	0.025	female
Malta	2008	2017	2.3	1.53	3.08	< 0.001	female
Netherlands	2009	2018	-0.42	-1.21	0.39	0.308	female
Poland	2009	2018	1.5	0.87	2.14	< 0.001	female
Portugal	2009	2018	2.35	1.86	2.83	< 0.001	female
Russian Federation	2010	2019	0.14	-0.11	0.4	0.253	female
Slovenia	2010	2019	2.46	2.21	2.7	< 0.001	female
Spain	2008	2017	3.94	3.25	4.64	< 0.001	female
Switzerland	2008	2017	0.15	-0.48	0.77	0.631	female
United Kingdom	2007	2016	-0.8	-1.09	-0.5	< 0.001	female
Denmark	2009	2018	-2.28	-3.24	-1.31	< 0.001	female
Faroe Islands	2009	2018	2.11	0.31	3.94	0.023	female
Finland	2009	2018	0.55	-1.07	2.19	0.51	female
Greenland	2009	2018	-0.31	-1.6	1	0.633	female
Iceland	2009	2018	-3.53	-6.64	-0.31	0.032	female
Norway	2009	2018	-1.03	-1.78	-0.27	0.009	female
Sweden	2009	2018	-1.06	-1.47	-0.64	< 0.001	female
North America							
Canada	2010	2019	-2.79	-3.47	-2.1	< 0.001	female
USA	2010	2019	-3.25	-3.47	-3.02	< 0.001	female
USA (White)	2010	2019	-3.13	-3.36	-2.91	< 0.001	female
USA (Black)	2010	2019	-3.99	-4.58	-3.41	< 0.001	female
South America							
Brazil	2010	2019	1.11	0.63	1.6	< 0.001	female
Chile	2009	2018	0.46	-1.96	2.94	0.712	female
Colombia	2008	2017	-1.32	-1.79	-0.84	< 0.001	female
Costa Rica	2010	2019	0.73	-2.29	3.84	0.641	female
Ecuador	2008	2017	0.27	-0.35	0.9	0.386	female
Oceania							
Australia	2009	2018	-1.39	-2.02	-0.75	< 0.001	female
New Zealand	2007	2016	-0.15	-0.42	0.11	0.239	female

CI: Confidence Interval; AAPC: average annual percent change.

Table S3. The AAPC of the incidence of tracheal, bronchus, and lung cancer in individuals of all ages.

Country	Lower endpoint	Upper endpoint	AAPC	95%CI-Low	95%CI-High	P-value	Sex
Africa							
Uganda*	2003	2012	-1.41	-6.23	3.67	0.56	male
Asia							
Bahrain	2003	2012	-6.53	-8.54	-4.49	< 0.001	male
China*	2003	2012	-1.78	-2.39	-1.16	< 0.001	male
Hong Kong, China*	2003	2012	-2.58	-2.84	-2.32	< 0.001	male
India*	2003	2012	-0.14	-2.29	2.05	0.897	male
Israel	2003	2012	-0.82	-1.77	0.14	0.093	male
Japan*	2001	2010	0.55	0.15	0.96	0.008	male
South Korea*	2003	2012	-1.35	-1.67	-1.03	< 0.001	male
Turkey*	2003	2012	-1.23	-2.76	0.32	0.118	male
Kuwait	2003	2012	-2.44	-4.68	-0.14	0.039	male
Philippines*	2003	2012	-4.69	-7.42	-1.89	0.001	male
Thailand*	2003	2012	-0.25	-0.67	0.18	0.233	male
Europe							
Austria	2003	2012	-1.37	-1.63	-1.11	< 0.001	male
Belarus	2003	2012	-0.79	-1.16	-0.42	< 0.001	male
Bulgaria	2003	2012	0.21	-0.26	0.67	0.352	male
Croatia	2003	2012	-2.71	-3.56	-1.85	< 0.001	male
Cyprus	2003	2012	2.14	-2.29	6.77	0.349	male
Czech Republic	2003	2012	-2.35	-2.54	-2.15	< 0.001	male
Estonia	2003	2012	-1.62	-1.88	-1.36	< 0.001	male
France*	2002	2011	0.12	-0.13	0.37	0.321	male
Germany*	2003	2012	-1.59	-2.09	-1.08	< 0.001	male
Ireland	2003	2012	-0.81	-1.09	-0.53	< 0.001	male
Italy*	2001	2010	-3.21	-3.53	-2.88	< 0.001	male
Lithuania	2003	2012	-1.61	-1.96	-1.26	< 0.001	male
Malta	2003	2012	-1.79	-2.72	-0.85	0.001	male
Netherlands	2003	2012	-1.76	-2.5	-1.01	< 0.001	male
Poland*	2003	2012	-2.85	-3.4	-2.3	< 0.001	male
Slovenia	2003	2012	3.31	3.06	3.57	< 0.001	male
Spain*	2001	2010	-0.34	-0.53	-0.15	0.002	male
Switzerland*	2003	2012	-2.08	-2.35	-1.81	< 0.001	male
United Kingdom*	2003	2012	-0.86	-1.19	-0.53	< 0.001	male
Denmark	2009	2018	-1.27	-1.34	-1.19	< 0.001	male

Faroe Islands	2009	2018	5.06	0.75	9.56	0.022	male
Finland	2009	2018	-1.82	-2.2	-1.45	< 0.001	male
Greenland	2009	2018	-2.82	-4.84	-0.75	0.009	male
Iceland	2009	2018	-4.24	-8.92	0.68	0.09	male
Norway	2009	2018	-1.45	-1.95	-0.94	< 0.001	male
Sweden	2009	2018	-1.1	-1.2	-0.99	< 0.001	male
North America							
Canada*	2003	2012	-2.18	-2.27	-2.09	< 0.001	male
USA*	2009	2018	-3.06	-3.32	-2.79	< 0.001	male
USA (White)*	2009	2018	-2.87	-3.02	-2.71	< 0.001	male
USA (Black)*	2009	2018	-3.76	-4.24	-3.28	< 0.001	male
South America							
Brazil*	2003	2012	-4.61	-8.69	-0.35	0.034	male
Chile*	2003	2012	-1.48	-4.49	1.62	0.317	male
Colombia*	2003	2012	-5.69	-8.21	-3.11	< 0.001	male
Costa Rica	2002	2011	-1.23	-1.8	-0.66	< 0.001	male
Ecuador*	2003	2012	-0.74	-1.67	0.21	0.122	male
Oceania							
Australia	2003	2012	-1.78	-1.99	-1.57	< 0.001	male
New Zealand	2003	2012	-2.5	-2.7	-2.29	< 0.001	male
Africa							
Uganda*	2003	2012	-2.14	-7.86	3.92	0.454	female
Asia							
Bahrain	2003	2012	-5.72	-9.61	-1.65	0.01	female
China*	2003	2012	-0.16	-0.75	0.43	0.571	female
Hong Kong, China	2003	2012	-1.09	-1.46	-0.72	< 0.001	female
India*	2003	2012	4.34	3.32	5.36	< 0.001	female
Israel	2003	2012	2.06	1.68	2.43	< 0.001	female
Japan*	2001	2010	2.32	1.55	3.1	< 0.001	female
South Korea*	2003	2012	2.35	1.79	2.9	< 0.001	female
Turkey*	2003	2012	0.99	-0.48	2.48	0.188	female
Kuwait	2003	2012	-4.43	-8.55	-0.13	0.045	female
Philippines*	2003	2012	-3.21	-5.6	-0.76	0.01	female
Thailand*	2003	2012	-0.8	-1.27	-0.33	0.002	female
Europe							
Austria	2003	2012	2.9	2.52	3.3	< 0.001	female

Belarus	2003	2012	1.62	0.68	2.58	0.002	female
Bulgaria	2003	2012	2.8	2.1	3.51	< 0.001	female
Croatia	2003	2012	2.41	1.79	3.03	< 0.001	female
Cyprus	2003	2012	4.67	2.22	7.18	0.001	female
Czech Republic	2003	2012	2.17	1.85	2.5	< 0.001	female
Estonia	2003	2012	1.36	0.93	1.79	< 0.001	female
France*	2002	2011	5.7	4.87	6.53	< 0.001	female
Germany*	2003	2012	2.37	1.7	3.04	< 0.001	female
Ireland	2003	2012	2.23	1.93	2.54	< 0.001	female
Italy*	2001	2010	1.34	0.62	2.06	0.002	female
Lithuania	2003	2012	0.62	0.16	1.08	0.01	female
Malta	2003	2012	9.39	6.43	12.43	< 0.001	female
Netherlands	2003	2012	3.41	2.81	4.01	< 0.001	female
Poland*	2003	2012	2.43	0.96	3.93	0.003	female
Slovenia	2003	2012	-1.92	-2.35	-1.49	< 0.001	female
Spain*	2001	2010	5.5	4.95	6.04	< 0.001	female
Switzerland*	2003	2012	2.2	1.7	2.69	< 0.001	female
United Kingdom*	2003	2012	2.01	1.74	2.28	< 0.001	female
Denmark	2009	2018	-0.16	-0.63	0.31	0.499	female
Faroe Islands	2009	2018	1.73	0.66	2.82	0.002	female
Finland	2009	2018	1.98	1.84	2.12	< 0.001	female
Greenland	2009	2018	1.18	0.14	2.23	0.027	female
Iceland	2009	2018	-2.71	-6.58	1.33	0.186	female
Norway	2009	2018	1.22	0.57	1.87	< 0.001	female
Sweden	2009	2018	0.3	-0.33	0.92	0.344	female
North America							
Canada*	2003	2012	-0.32	-0.81	0.17	0.202	female
USA*	2009	2018	-1.79	-2	-1.58	< 0.001	female
USA (White)*	2009	2018	-1.84	-2.1	-1.57	< 0.001	female
USA (Black)*	2009	2018	-2.12	-2.75	-1.48	< 0.001	female
South America							
Brazil*	2003	2012	2.43	1.01	3.88	0.002	female
Chile*	2003	2012	0.12	-4.3	4.73	0.956	female
Colombia*	2003	2012	-0.62	-1.19	-0.05	0.033	female
Costa Rica	2002	2011	0.28	-0.57	1.14	0.503	female
Ecuador*	2003	2012	2.13	1.05	3.22	< 0.001	female

Oceania							
Australia	2003	2012	1.47	1.23	1.7	< 0.001	female
New Zealand	2003	2012	1.05	0.79	1.31	< 0.001	female

*Subnational data; CI: Confidence Interval; AAPC: average annual percent change.

Table S4. The AAPC of the incidence of tracheal, bronchus, and lung cancer in individuals older than 50 years old.

Country	Lower endpoint	Upper endpoint	AAPC	95%CI-Low	95%CI-High	P-value	Sex
Africa							
Uganda*	2003	2012	-3	-8.7	3.06	0.302	male
Asia							
Bahrain	2003	2012	-6.54	-8.58	-4.45	< 0.001	male
China*	2003	2012	-1.7	-2.43	-0.96	< 0.001	male
Hong Kong, China*	2003	2012	-2.6	-2.88	-2.32	< 0.001	male
India*	2003	2012	0.11	-2.04	2.32	0.918	male
Japan*	2001	2010	0.7	0.24	1.17	0.003	male
South Korea*	2003	2012	-1.32	-1.65	-0.99	< 0.001	male
Turkey*	2003	2012	-0.73	-2.31	0.88	0.374	male
Kuwait	2003	2012	-2.06	-4.44	0.37	0.089	male
Philippines*	2003	2012	-3.05	-3.71	-2.38	< 0.001	male
Thailand*	2003	2012	-0.06	-0.5	0.4	0.797	male
Europe							
Austria	2003	2012	-1.17	-1.45	-0.89	< 0.001	male
Belarus	2003	2012	-0.69	-1.08	-0.31	0.001	male
Bulgaria	2003	2012	0.62	0.1	1.15	0.022	male
Croatia	2003	2012	-0.85	-1.33	-0.36	0.002	male
Cyprus	2003	2012	2.2	-0.54	5.02	0.116	male
Czech Republic	2003	2012	-2	-2.21	-1.79	< 0.001	male
Estonia	2003	2012	-1.36	-1.61	-1.1	< 0.001	male
France*	2002	2011	0.49	0.22	0.77	0.002	male
Germany*	2003	2012	-1.24	-1.76	-0.72	< 0.001	male
Ireland	2003	2012	-0.86	-1.14	-0.58	< 0.001	male
Italy*	2001	2010	-3.1	-3.39	-2.81	< 0.001	male
Lithuania	2003	2012	-1.38	-1.73	-1.02	< 0.001	male
Malta	2003	2012	-1.6	-2.54	-0.66	0.002	male
Netherlands	2003	2012	-1.76	-2.43	-1.08	< 0.001	male
Poland*	2003	2012	-2.57	-3.16	-1.97	< 0.001	male
Slovenia	2003	2012	-2.5	-3.97	-1	0.001	male
Spain*	2001	2010	0.02	-0.16	0.21	0.778	male
Switzerland*	2003	2012	-1.91	-2.25	-1.58	< 0.001	male
United Kingdom*	2003	2012	-0.84	-1.16	-0.51	< 0.001	male
Denmark	2010	2019	-1.21	-1.29	-1.13	< 0.001	male

Faroe Islands	2010	2019	3.05	-0.76	7.02	0.116	male
Finland	2010	2019	-1.97	-2.3	-1.65	< 0.001	male
Greenland	2010	2019	-2.89	-4.76	-0.98	0.004	male
Iceland	2010	2019	-4.06	-7.73	-0.25	0.037	male
Norway	2010	2019	-1.57	-2.08	-1.06	< 0.001	male
Sweden	2010	2019	-2.31	-3.32	-1.3	< 0.001	male
North America							
Canada*	2003	2012	-2.11	-2.2	-2.01	< 0.001	male
USA*	2009	2018	-2.95	-3.22	-2.69	< 0.001	male
USA (White)*	2009	2018	-2.78	-2.94	-2.62	< 0.001	male
USA (Black)*	2009	2018	-3.6	-4.1	-3.09	< 0.001	male
South America							
Brazil*	2003	2012	-4.7	-8.5	-0.75	0.02	male
Chile*	2003	2012	-0.92	-4.05	2.32	0.546	male
Colombia*	2003	2012	-5.46	-8.21	-2.62	< 0.001	male
Costa Rica	2002	2011	-1.21	-1.81	-0.62	< 0.001	male
Ecuador*	2003	2012	-0.83	-1.75	0.1	0.079	male
Oceania							
Australia	2003	2012	-1.83	-2.04	-1.62	< 0.001	male
New Zealand	2003	2012	-2.5	-2.71	-2.29	< 0.001	male
Africa							
Uganda*	2003	2012	-1.78	-7.38	4.17	0.525	female
Asia							
Bahrain	2003	2012	-5.65	-9.37	-1.78	0.008	female
China*	2003	2012	-0.29	-0.95	0.37	0.36	female
Hong Kong, China	2003	2012	-1.15	-1.55	-0.76	< 0.001	female
India*	2003	2012	4.31	3.2	5.43	< 0.001	female
Japan*	2001	2010	1.92	1.33	2.52	< 0.001	female
South Korea*	2003	2012	2.37	1.76	2.98	< 0.001	female
Turkey*	2003	2012	1.14	-0.35	2.66	0.135	female
Kuwait	2003	2012	-4.23	-8.54	0.29	0.064	female
Philippines*	2003	2012	-1.18	-1.63	-0.73	< 0.001	female
Thailand*	2003	2012	-0.71	-1.2	-0.21	0.008	female
Europe							
Austria	2003	2012	3.41	3.05	3.78	< 0.001	female
Belarus	2003	2012	1.55	0.5	2.62	0.006	female

Bulgaria	2003	2012	3.02	2.19	3.86	< 0.001	female
Croatia	2003	2012	2.66	2.1	3.22	< 0.001	female
Cyprus	2003	2012	4.69	2.22	7.22	0.001	female
Czech Republic	2003	2012	2.65	2.29	3	< 0.001	female
Estonia	2003	2012	1.44	1	1.89	< 0.001	female
France*	2002	2011	6.83	5.94	7.72	< 0.001	female
Germany*	2003	2012	2.98	2.34	3.63	< 0.001	female
Ireland	2003	2012	2.29	1.95	2.64	< 0.001	female
Italy*	2001	2010	1.73	1.03	2.43	< 0.001	female
Lithuania	2003	2012	0.4	-0.08	0.89	0.099	female
Malta	2003	2012	10.09	6.37	13.95	< 0.001	female
Netherlands	2003	2012	4.24	3.74	4.75	< 0.001	female
Poland*	2003	2012	3.41	1.85	4.99	< 0.001	female
Slovenia	2003	2012	3.5	3.21	3.79	< 0.001	female
Spain*	2001	2010	7.95	6.44	9.49	< 0.001	female
Switzerland*	2003	2012	2.69	2.32	3.07	< 0.001	female
United Kingdom*	2003	2012	2.3	1.62	2.99	< 0.001	female
Denmark	2010	2019	-0.46	-1.1	0.19	0.164	female
Faroe Islands	2010	2019	1.88	0.81	2.97	0.001	female
Finland	2010	2019	2.4	2.21	2.6	< 0.001	female
Greenland	2010	2019	0.32	-0.75	1.39	0.554	female
Iceland	2010	2019	-2.2	-5.23	0.93	0.166	female
Norway	2010	2019	1.33	0.75	1.91	< 0.001	female
Sweden	2010	2019	-0.08	-0.79	0.63	0.814	female
North America							
Canada*	2003	2012	-0.02	-0.51	0.48	0.947	female
USA*	2009	2018	-1.61	-1.82	-1.39	< 0.001	female
USA (White)*	2009	2018	-1.68	-1.96	-1.41	< 0.001	female
USA (Black)*	2009	2018	-1.55	-2.15	-0.96	< 0.001	female
South America							
Brazil*	2003	2012	2.13	0.62	3.66	0.008	female
Chile*	2003	2012	-0.06	-4.85	4.98	0.98	female
Colombia*	2003	2012	-0.64	-1.19	-0.09	0.024	female
Costa Rica	2002	2011	0.42	-0.49	1.33	0.352	female
Ecuador*	2003	2012	2.15	1.01	3.29	0.001	female
Oceania							

Australia	2003	2012	1.57	1.32	1.81	< 0.001	female
New Zealand	2003	2012	0.95	0.68	1.22	< 0.001	female

*Subnational data; CI: Confidence Interval; AAPC: average annual percent change.

Table S5. The AAPC of the incidence of tracheal, bronchus, and lung cancer in individuals younger than 50 years old.

Country	Lower endpoint	Upper endpoint	AAPC	95%CI-Low	95%CI-High	P-value	Sex
Africa							
Uganda*	2003	2012	-1.12	-5.35	3.29	0.592	male
Asia							
China*	2003	2012	-2.95	-4.72	-1.15	0.001	male
Hong Kong, China*	2003	2012	-3.42	-3.82	-3.01	< 0.001	male
India*	2003	2012	-0.86	-1.79	0.08	0.071	male
Japan*	2001	2010	-1.66	-2.9	-0.41	0.014	male
South Korea*	2003	2012	-1.94	-2.9	-0.96	0.001	male
Turkey*	2003	2012	-6.4	-9.15	-3.56	< 0.001	male
Thailand*	2003	2012	-2.15	-3.34	-0.95	0.002	male
Europe							
Austria	2003	2012	-3.59	-4.67	-2.49	< 0.001	male
Belarus	2003	2012	-2.28	-2.76	-1.8	< 0.001	male
Bulgaria	2003	2012	-2.94	-3.87	-2	< 0.001	male
Croatia	2003	2012	-7.02	-8.9	-5.11	< 0.001	male
Cyprus	2002	2012	0.08	-3.2	3.47	0.959	male
Czech Republic	2003	2012	-8.22	-9.01	-7.43	< 0.001	male
Estonia	2003	2012	-6.78	-8.67	-4.85	< 0.001	male
France*	2002	2011	-4.33	-5.71	-2.92	< 0.001	male
Germany*	2003	2012	-6.22	-7.22	-5.21	< 0.001	male
Ireland	2003	2012	0.24	-1.19	1.69	0.731	male
Italy*	2001	2010	-5.15	-6.91	-3.36	< 0.001	male
Lithuania	2003	2012	-4.61	-5.46	-3.74	< 0.001	male
Malta	2003	2012	-4.38	-8.86	0.31	0.065	male
Netherlands	2003	2012	-2.56	-2.91	-2.22	< 0.001	male
Poland*	2003	2012	-6.46	-8.53	-4.34	< 0.001	male
Slovenia	2003	2012	-6.69	-9.52	-3.76	< 0.001	male
Spain*	2001	2010	-6.18	-9.91	-2.3	0.002	male
Switzerland*	2003	2012	-4.27	-6.06	-2.44	< 0.001	male
United Kingdom*	2003	2012	-2.45	-2.89	-2	< 0.001	male
Denmark	2010	2019	-4.31	-5.5	-3.1	< 0.001	male
Finland	2010	2019	-3.62	-3.86	-3.39	< 0.001	male
Greenland	2007	2019	-2.63	-4.46	-0.77	0.007	male
Iceland	2009	2019	-10.76	-19.12	-1.54	0.024	male
Norway	2010	2019	-4.27	-5.46	-3.07	< 0.001	male

Sweden	2010	2019	-3.06	-3.39	-2.74	< 0.001	male
North America							
Canada*	2003	2012	-3.47	-3.69	-3.26	< 0.001	male
USA*	2009	2018	-4.84	-5.29	-4.39	< 0.001	male
USA (White)*	2009	2018	-5.68	-6.74	-4.61	< 0.001	male
USA (Black)*	2009	2018	-5.84	-6.48	-5.2	< 0.001	male
South America							
Brazil*	2003	2012	-1.59	-4.54	1.45	0.284	male
Colombia	2003	2012	-11.71	-20.34	-2.14	0.018	male
Costa Rica	2002	2011	-1.61	-2.9	-0.31	0.017	male
Ecuador	2003	2012	0.31	-2.32	3.01	0.812	male
Oceania							
Australia	2003	2012	-0.93	-1.62	-0.24	0.011	male
New Zealand	2003	2012	-1.82	-2.72	-0.9	< 0.001	male
Africa							
Uganda*	2003	2012	-5.65	-12.56	1.81	0.125	female
Asia							
China*	2003	2012	0.99	0.26	1.73	0.011	female
Hong Kong, China	2003	2012	-1.55	-2	-1.1	< 0.001	female
India*	2003	2012	3.5	1.64	5.39	0.001	female
Japan*	2001	2010	1.39	-1	3.83	0.23	female
South Korea*	2003	2012	2.02	0.66	3.41	0.007	female
Turkey*	2003	2012	2.34	-0.41	5.17	0.089	female
Thailand*	2003	2012	-1.6	-2.56	-0.64	0.003	female
Europe							
Austria	2003	2012	-0.12	-1.27	1.04	0.821	female
Belarus	2003	2012	1.65	1.03	2.28	< 0.001	female
Bulgaria	2003	2012	1.78	0.95	2.63	< 0.001	female
Croatia	2003	2012	1.09	-0.15	2.36	0.083	female
Cyprus	2002	2012	2.21	-3.16	7.88	0.395	female
Czech Republic	2003	2012	-2.34	-3.26	-1.4	< 0.001	female
Estonia	2003	2012	0.64	-0.63	1.92	0.314	female
France*	2002	2011	1.75	-4.41	8.3	0.587	female
Germany*	2003	2012	-1.47	-3.23	0.33	0.1	female
Ireland	2003	2012	-2.8	-5.51	-0.01	0.049	female
Italy*	2001	2010	-1.34	-3.56	0.93	0.218	female

Lithuania	2003	2012	2.08	0.41	3.78	0.017	female
Malta	2003	2012	5.79	2	9.73	0.005	female
Netherlands	2003	2012	-2.28	-3.6	-0.95	0.001	female
Poland*	2003	2012	-9.35	-15.08	-3.23	0.003	female
Slovenia	2003	2012	-2.92	-4.91	-0.9	0.007	female
Spain*	2001	2010	-0.77	-4.92	3.55	0.721	female
Switzerland*	2003	2012	-1.46	-3.97	1.11	0.238	female
United Kingdom*	2003	2012	-0.94	-1.49	-0.4	0.002	female
Denmark	2010	2019	-4.51	-6.12	-2.89	< 0.001	female
Finland	2010	2019	-1.48	-2.64	-0.31	0.014	female
Greenland	2006	2018	-2.42	-4.03	-0.79	0.005	female
Iceland	2009	2019	-4.92	-7.02	-2.78	< 0.001	female
Norway	2010	2019	-1.47	-2.35	-0.57	0.002	female
Sweden	2010	2019	-2.45	-3.06	-1.85	< 0.001	female
North America							
Canada*	2003	2012	-4.56	-6.84	-2.22	< 0.001	female
USA*	2009	2018	-4.45	-5.27	-3.62	< 0.001	female
USA (White)*	2009	2018	-4.25	-4.96	-3.54	< 0.001	female
USA (Black)*	2009	2018	-8.85	-11.81	-5.8	< 0.001	female
South America							
Brazil*	2003	2012	4.42	1.16	7.79	0.01	female
Colombia	2003	2012	-1.21	-2.71	0.31	0.112	female
Costa Rica	2002	2011	-0.94	-2.32	0.45	0.175	female
Ecuador	2003	2012	1.37	-0.69	3.47	0.185	female
Oceania							
Australia	2003	2012	0.52	-0.23	1.27	0.162	female
New Zealand	2003	2012	-0.23	-1.07	0.62	0.585	female

*Subnational data; CI: Confidence Interval; AAPC: average annual percent change.
