# Supplementary material for: Worldwide burden and epidemiological trends of tracheal, bronchus, and lung cancer: A population-based study
Source: eBioMedicine. 2022 Mar 18;78:103951. doi: 10.1016/j.ebiom.2022.103951 (PMC8935504; doi:10.1016/j.ebiom.2022.103951)
Supplement: Supplementary file 2 [file mmc2.docx]

Caption for supplementary material

**Figure S1 The average annual percentage change (AAPC) of the incidence of tracheal, bronchus, and lung cancer in men and women older than 50 years old.** The AAPC is denoted by the coloured bars and 95% confidence intervals (CIs) are represented by error bars. The permutation test is applied for testing between two different joinpoint models. *Subnational data.

**Figure S2 The average annual percentage change (AAPC) of the incidence of tracheal, bronchus, and lung cancer in men and women younger than 50 years old.** The AAPC is denoted by the coloured bars and 95% confidence intervals (CIs) are represented by error bars. The permutation test is applied for testing between two different joinpoint models. *Subnational data.

**Table S1 The incidence and mortality data of tracheal, bronchus, and lung cancer.**

**Table S2 The AAPC of the mortality of tracheal, bronchus, and lung cancer in individuals of all ages.**

**Table S3 The AAPC of the incidence of tracheal, bronchus, and lung cancer in individuals of all ages.**

**Table S4 The AAPC of the incidence of tracheal, bronchus, and lung cancer in individuals older than 50 years old.**

**Table S5 The AAPC of the incidence of tracheal, bronchus, and lung cancer in individuals younger than 50 years old.**
